# Prodigal: prokaryotic gene recognition and translation initiation site identification

**DOI:** 10.1186/1471-2105-11-119

**Published:** 2010-03-08

**Authors:** Doug Hyatt, Gwo-Liang Chen, Philip F LoCascio, Miriam L Land, Frank W Larimer, Loren J Hauser

**Affiliations:** 1 Computational Biology and Bioinformatics Group, Oak Ridge National Laboratory, Oak Ridge, TN 37831, USA; 2 Genome Science and Technology Graduate School, The University of Tennessee, Knoxville, TN 37996, USA; 3 DOE Joint Genome Institute, Oak Ridge National Laboratory, Oak Ridge TN 37831, USA

## Abstract

**Background:**

The quality of automated gene prediction in microbial organisms has improved steadily over the past decade, but there is still room for improvement. Increasing the number of correct identifications, both of genes and of the translation initiation sites for each gene, and reducing the overall number of false positives, are all desirable goals.

**Results:**

With our years of experience in manually curating genomes for the Joint Genome Institute, we developed a new gene prediction algorithm called Prodigal (PROkaryotic DYnamic programming Gene-finding ALgorithm). With Prodigal, we focused specifically on the three goals of improved gene structure prediction, improved translation initiation site recognition, and reduced false positives. We compared the results of Prodigal to existing gene-finding methods to demonstrate that it met each of these objectives.

**Conclusion:**

We built a fast, lightweight, open source gene prediction program called Prodigal http://compbio.ornl.gov/prodigal/. Prodigal achieved good results compared to existing methods, and we believe it will be a valuable asset to automated microbial annotation pipelines.

## Background

Microbial gene prediction is a well studied, and some would say solved, problem, but the truth is that there is still much room for improvement, especially in understanding how translation initiation mechanisms work in prokaryotes. Existing methods for bacterial and archaeal gene prediction include the popular Glimmer [1] and GenemarkHMM [2] packages, both of which are included at NCBI alongside Genbank [3] annotations (Prodigal is also included), as well as other methods such as Easygene [4] and MED [5].

Current gene recognition methods perform relatively well in low GC content genomes, but the accuracy drops considerably in high GC content genomes. High GC genomes contain fewer overall stop codons and more spurious open reading frames (ORFs). These false ORFs are often selected by programs instead of real ORFs in the same genomic region. In addition, the longer ORFs in high GC genomes contain more potential start codons, thus leading to a drop in accuracy of the translation initiation site (or TIS) predictions as well.

Translation initiation site prediction in existing microbial gene-finding tools has not proven to be sufficiently adequate, and this has motivated a number of tools to be developed specifically to correct the start calls of current methods. These tools include GSFinder [6], TiCO [7], and TriTISA [8]. It is our view that a single gene prediction algorithm should be able to match the performance of the above methods, rather than needing to run two programs to attain the desired level of accuracy in start predictions.

Finally, most methods tend to predict too many genes. Although many of the short genes predicted by current programs that have no existing BLAST [9] hits might be real, the likelihood is that most are false positives. We base this assertion on the fact that genome-wide proteomics studies that search the entire set of all potential ORFs do not identify a significant number of peptides in these genes [10]. In the construction of a novel algorithm, we determined it would be preferable to sacrifice some genuine predictions if it meant also eliminating a much larger number of false identifications.

With the advent of faster sequencing technologies, it is likely that in the future less time will be spent on finishing microbial genome sequence. It is also likely that researchers will not often be able to curate manually the gene predictions delivered by automated pipelines. It is therefore important to improve the current methodologies to obtain higher quality gene predictions, better translation initiation site predictions, and a reduction in the number of false positives.

## Implementation

To address these challenges, we constructed a novel gene-finding algorithm called Prodigal. In designing the Prodigal algorithm, we decided to use a "trial and error" approach. We began by building a set of curated genomes that had been analyzed using the JGI ORNL pipeline http://genome.ornl.gov/. This pipeline consisted of a combination of Critica [11] and Glimmer [1], BLAST [9] to locate missing genes and correct errors, and a final round of manual expert curation. To this initial set of ten genomes we added *Escherichia coli K12 *(both the Genbank file and the Ecogene Verified Protein Starts data set [12]), *Bacillus subtilis*, and *Pseudomonas aeruginosa*. With these sets in hand, it became possible to validate or exclude changes to the algorithm based on whether or not the performance on the test set of genes increased or decreased, respectively. In the final stages of validating the rules in the program, we expanded this set to include over 100 genomes from Genbank.

It should be noted that we only used this set to determine very general rules about the nature of prokaryotic genes, such as gene size, maximum overlap between two genes (both on the same strand and on opposite strands), and RBS motif usage. In addition, we tuned several constants in the program based on performance on this data set. This set was also used to exclude ideas that caused deterioration in performance across many genomes. (These failed ideas are too numerous to include in this publication). Because we intended to validate Prodigal's performance by examining *E. coli, B. subtilis*, and *P. aeruginosa*, we also verified that each of these decisions we made also maximized performance on the remaining genomes in our set. Changes were not retained if they were merely "local" improvements to a subset of genomes, especially not genomes on which we intended to test the program's performance.

In order for Prodigal to run in a completely unsupervised fashion, it needed to be able to learn all the necessary properties of the input organism, including start codon usage (ATG vs. GTG vs. TTG), ribosomal binding site (RBS) motif usage, GC frame plot bias, hexamer coding statistics, and other information necessary to build a complete training profile. To gather statistics from a finished sequence or set of sequences, the algorithm first had to determine automatically a set of putative "real" genes on which to train.

Prodigal constructs its training set of genes by examining the GC frame plot in the ORFs in the genome. The program begins by traversing the entire input sequence and examining the bias for G's and C's in each of the three codon positions in each open reading frame. The highest GC content codon position for an ORF is considered the "winner", and a running sum for that codon position is incremented. Once all ORFs have been processed, the sums give an approximate measure of the preference of each codon position for G and C. The values for each codon position are normalized around 1 and divided by 1/3. If 2/3 of the codons in ORFs prefer G or C in the third position, for example, then the bias score for that position would be 2. We tried converting this bias to a log score, but this was found to decrease the quality of the results.

Using this GC bias information, Prodigal constructs preliminary coding scores for each gene in the genome. This is done by multiplying the relative codon bias for each of the three positions by the number of codons in the putative gene in which that codon position is the maximal GC frame (in the 120 bp window centered on that position). We chose 120 bp for the window size because that is the default window size for GC frame plot calculation in Artemis [13], and, in the experience of our manual curators, this default was an optimal setting. So, for example, if an entire gene contains the most G's and C's in its third codon position, the score for that gene would be the length of the gene multiplied by our codon bias score for frame 3. If instead this gene is too long, then the frame plot information should change in the spurious upstream region. These bases would be multiplied by a lower GC frame bias score (for example, for frame 2, which is seldom the highest GC content frame in real genes). The score S for a given gene starting at location n1 and ending at location n2 can be given by:

where B(i) is the bias score for codon position *i*, and l(i) is the number of bases in the gene where the 120 bp maximal window at that position corresponds to codon position *i*.

With this preliminary coding score measure based on simple GC codon position statistics, Prodigal scores every start-stop pair above 90 bp in the entire genome. (We tried allowing genes smaller than this, but the number of false positives became problematic.) Prodigal then performs a dynamic programming [14] across the whole sequence (or set of sequences) to identify a maximal "tiling path" of genes to train on. The purpose of this dynamic programming method is to force the program to choose between two heavily overlapping ORFs in the same genomic context. In theory, one of these ORFs should match the preferred GC codon position of the organism, whereas the other one should not.

**Figure 1:**
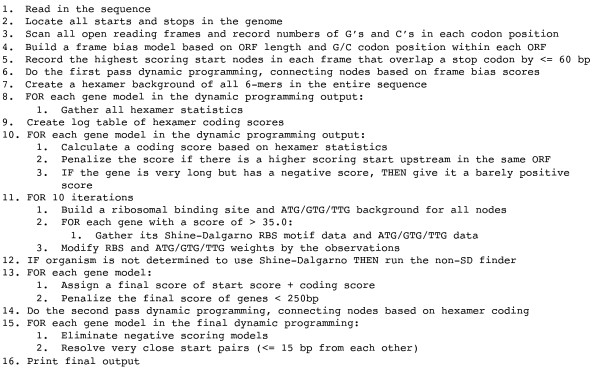
**Pseudocode description of the Prodigal algorithm**.

Prodigal utilizes the same dynamic programming algorithm both for its preliminary training phase and for its final gene calling phase. Each node in the dynamic programming matrix is either a start codon (ATG, GTG, or TTG only: the program does not consider nonstandard starts such as ATA, ATT, or CTG) or a valid stop codon (specified by the translation table code). In addition, start and stop nodes are added in each frame at the edges of the sequence to handle cases where genes run off the edge of contigs, a common occurrence in draft and metagenomic sequence data. The connection of a start node to its corresponding stop node represents a gene, whereas the connection of a 3' end to a new 5' end represents intergenic space. The score of a "gene" connection is the precalculated coding score for that gene, whereas the score for an intergenic connection is a small bonus or penalty based on the distance between the two genes. Figure 1 illustrates these dynamic programming connections in action.

Since dynamic programming cannot go backwards (a partial solution to a given point must also be a part of the final solution, prohibiting the concept of past information suddenly changing), we need a special set of rules to handle overlapping genes. Prodigal accomplishes this by pre-calculating the best overlapping genes in all three frames for each 3' end in the genome. So, for example, for a stop codon at position 15,000, the program would look 60 bp upstream of position 15,000 and locate the highest scoring overlapping gene in each frame (there may not be one). With this information in hand, a new type of connection can be established, that of a 3' end of one gene to a 3' end of a second gene on the same strand. In this case, the 5' end of the second gene is *implied *by the connection, since the best start has already been calculated. A maximal overlap of 60 bp is allowed between two genes on the same strand. For opposite strand overlap, we allow 200 bp overlap between 3' ends of genes, but 5' ends of genes are not permitted to overlap. These connections are represented by the 3' end of a forward gene connecting to the 5' end of a reverse gene, wherein the 3' end of the second gene is implied (there can be only one stop codon for a given start). These overlap values were determined by recording overlaps between genes in the Genbank files of our test set. Although we may merely be encouraging Prodigal's overlap rules to be similar to previous gene predictors, our manual curators also felt these were reasonable values for overlap based on their experience examining finished genomes. Table 1 shows a summary of the different types of dynamic programming connections allowed in Prodigal.

**Table 1:** Dynamic Programming Connections in Prodigal

Left Node	Right Node	Connection Type	Connection Score
5' forward	3' forward	Gene	Start+coding score

3' reverse	5' reverse	Gene	Start+coding score

3' forward	5' forward	Intergenic Space	Distance modifiers

3' forward	3' reverse	Intergenic Space	Distance modifiers

5' reverse	3' reverse	Intergenic Space	Distance modifiers

5' reverse	5' forward	Intergenic Space	Distance modifiers

3' forward	3' forward	Overlapping Genes	Score of 2^nd ^gene

3' reverse	3' reverse	Overlapping Genes	Score of 2^nd ^gene

3' forward	5' reverse	Opposite Strand Overlap	Score of 2^nd ^gene

Table 1 shows the types of dynamic programming connections in the algorithm. Each end of a gene is a node, and connections between these nodes represent either genes or the space between genes. The more complicated connections indicate overlapping genes.

Once the preliminary dynamic programming algorithm has completed, the next step is to gather statistics from the putative genes and construct a more rigorous coding scorer. Prodigal does this in a very simplistic way, by simply looking at in-frame hexamer coding frequencies for a gene relative to the background. A lookup table of 4096 values is created, one for each 6-mer, where the value of a given word *w *is:

where C is the coding score, G is the percentage occurrence of that word within our gene training set, and B is the percentage occurrence of that word across the entire sequence (irrespective of frames). So, for example, if a word is twice as likely to occur in a gene as it is in the background, the score for that word would be log(2). This corresponds to a 5^th^-order Markov model [1,2]. A floor and a ceiling are also established on this score to handle cases where there is insufficient data for a given word.

The final coding score for a gene beginning at position *n1 *and ending at position *n2 *can be written as

where S is the sum of the coding scores (*C*) for the in-frame hexamers (the set of words *w*) in the gene. In addition, Prodigal modifies this coding score based on information about what lies upstream of the selected start. For example, if a gene 1000..3000 has a score of 500.0, and the gene 1200..3000 has a score of 400.0, Prodigal modifies the score of the second gene to be 400-(500-400) = 300. The reason for this modification is to penalize choosing a truncated version of a gene when a longer, higher-scoring version of the same gene could also be chosen. In the dynamic programming model, this can be thought of as penalizing a connection to an interior start by subtracting the difference between the two potential genes. The purpose for this modification is to discourage the truncation of genes through choosing a gene on the opposite strand that overlaps with and erases the beginning of the longer version of the gene, a common occurrence in current gene-finders. In addition, Prodigal implements a few more minor tweaks to the coding score, including boosting the score of particularly long genes (dependent on the GC content of the organism: ~700 bp or so in low GC, ~1200 or so in high GC) to be minimally positive if the preliminary coding score is negative.

Once Prodigal has calculated coding potential scores for every start-stop pair in the genome, the next step is to create a translation initiation site scoring system from the training set. The program constructs a background of ATG, GTG, and TTG frequencies off all start nodes in the genome. It also builds a background of RBS motifs based on the Shine-Dalgarno sequence [15]. Unlike many methods, which use a position-weight matrix or Gibbs sampling method to find motifs, Prodigal begins by assuming that the SD motif will be utilized by the organism. If this turns out not to be the case, it runs a more rigorous motif finder. But, to start with, the program attempts to determine if the SD motif is widely utilized by the genome in question.

For RBS motifs, Prodigal utilizes a concept of bins, each of which corresponds to a set of RBS motifs and spacer distances (the spacer is the distance between the motif and the translation initiation codon). Table 2 shows the default priority of these bins, from lowest scoring to highest scoring.

**Table 2:** Shine-Dalgarno RBS Motifs in Prodigal

Bin #	RBS Motif	RBS Spacer
0	None	None

1	*GGA, GAG, AGG*	3-4 bp

2	*GGA, GAG, AGG, AGxAG, GGxGG*	13-15 bp

3	*AGGA, GGAG, GAGG, AGxAGG, AGGxGG*	13-15 bp

4	*AGxAG*	11-12 bp

5	*AGxAG*	3-4 bp

6	*GGA, GAG, AGG*	11-12 bp

7	*GGxGG*	11-12 bp

8	*GGxGG*	3-4 bp

9	*AGxAG*	5-10 bp

10	*AGGAG, GGAGG, AGGAGG*	13-15 bp

11	*AGGA, GGAG, GAGG*	3-4 bp

12	*AGGA, GGAG, GAGG*	11-12 bp

13	*GGA, GAG, AGG*	5-10 bp

14	*GGxGG*	5-10 bp

15	*AGGA*	5-10 bp

16	*GGAG, GAGG*	5-10 bp

17	*AGxAGG, AGGxGG*	11-12 bp

18	*AGxAGG, AGGxGG*	3-4 bp

19	*AGxAGG, AGGxGG*	5-10 bp

20	*AGGAG, GGAGG*	11-12 bp

21	*AGGAG*	3-4 bp

22	*AGGAG*	5-10 bp

23	*GGAGG*	3-4 bp

24	*GGAGG*	5-10 bp

25	*AGGAGG*	11-12 bp

26	*AGGAGG*	3-4 bp

27	*AGGAGG*	5-10 bp

Table 2 shows the default bins for the RBS motifs. An 'x' in the middle of a motif indicates a mismatch is allowed. The right column shows the spacer distance allowed between the translation start and the motif. The leftmost column indicates the initial "score" assigned to these bins, i.e. higher bins are better. In subsequent iterations, however, these values may change, and, in non-SD-using organisms, bin 0 (no RBS) may emerge as the highest scoring.

In the initial background, the motif in a higher numbered bin takes priority over one in a lower numbered bin if both are found upstream of a start site. These bins were rigorously determined by examining the detailed data set of curated Genbank files (and the EcoGene Verified Protein Starts [12]). Prodigal examines the initial coding peaks in every open reading frame (where the coding peak is the highest scoring start-stop pair for a given stop codon) with a coding score of 35.0 or higher (a somewhat arbitrary threshold chosen that would include only longer genes, which are more likely to be real). From these coding peaks, it builds a log-likelihood model similar to the coding score, described by:

where S is the score, R is the observed percentage of this type in our training set, and B is the percentage occurrence in the background. This method is used both for start codon usage (ATG, GTG, or TTG) as well as for the SD bin motif (from the table above). These scores are summed together and multiplied by a constant (4.25, corresponding to about 16 bp of coding score, determined empirically from maximal performance on our test set of genomes, and later verified on a larger set of genomes from Genbank), then added to the coding score. Prodigal goes through every start-stop node and performs this calculation, modifying the default coding score by the quality of its start codon information. This leads to a new set of "peaks" for the set of training ORFs. For example, an ATG with a slightly lower coding score than a TTG in the same ORF could overtake it with the additional start score added (assuming the organism uses ATG as a start codon more than TTG).

Once a new set of peaks has been determined, Prodigal reconstructs the background for both SD motif and start codon usage. In this iteration and in subsequent ones, it no longer assumes a higher numbered bin is better for RBS motifs, and it instead relies on the log likelihoods calculated in the previous iteration to find the best upstream motif for a given start site. Prodigal performs several iterations of this process, moving the peaks around based on subsequent information until they no longer move significantly. When the peaks no longer move, it determines the final set of weights based on statistics gathered from this final set of putative "real" start codons.

The end result is a set of log-likelihood weights for ATG/GTG/TTG information and for each of the above RBS bins. If the zero bin for RBS motifs, which corresponds to no SD motif, is positive, or if the zero bin is above -0.5 and the 4-base motif bins are less than 1.0, then Prodigal determines that this organism does not use the SD motif strongly, and it runs a more rigorous motif finder. In examining over 800 finished genomes in Genbank, we determined about 10% of them did not use the SD motif strongly. Most of these genomes were cyanobacteria, chlorobii, or archaea, which seem to use different translation mechanisms than the more common SD motif.

If it is found that the organism does not use the SD motif, Prodigal searches exhaustively for alternative motifs. It does so by looking at the occurrence of all 3-mer motifs in the initial set of peaks, and locating all 3-mers that occur in at least 20% of the high-scoring gene models. From these motifs, it then performs an iterative algorithm similar to the above. The bins instead correspond to every word of size 3-6 bp (mismatches allowed only in the center of 5-6 bp words, just as in the SD RBS motif table above) with every potential spacer size (3-4 bp, 5-10 bp, 11-12 bp, and 13-15 bp). All words 3-6 bp that do not occur frequently enough are combined in the "no RBS motif" bin. Prodigal then arrives at a similar set of weights for no RBS motif, as well as for each 3-6 bp motif that contains the commonly occurring 3 bp motif as a subset. In *Aeropyrum pernix*, a strong GGTG motif is located, whereas in many cyanobacteria, Prodigal latches onto AT-rich motifs like TATA and TAAA.

Finally, we added a scoring system to capture information in the regions outside those examined by the RBS scorer (1-2 bp and 15 bp to 45 bp upstream from the translation start site). This scoring system builds a position weight matrix on the whole region. Although this scoring system is very crude and captures only general characteristics (AT-richness, simple base preferences, etc.), it was found to be quite effective in some genomes. This generic upstream scoring system is not part of the iterative algorithm; the data is instead gathered from the final iteration of the start training.

Once Prodigal has start score weights for both start codon type (ATG/GTG/TTG) and RBS motif/spacer distance, it then scores every start node in the entire sequence. The final score for a start node is simply

in which S is the final score, R is the RBS motif score, T is the start type score, U is the upstream score, and C is the coding score. For the RBS weight, Prodigal uses the SD motif score if it determines that the organism uses Shine-Dalgarno, the secondary RBS motif score if it finds a clear-cut secondary motif, and the maximum of the two systems if neither system located a strong RBS motif. This latter method was shown to work well in some genomes such as cyanobacteria and crenarchaea that tended to have AT-rich upstream regions but still occasionally used the SD motif for some genes (such as ribosomal proteins).

A linear combination of the various elements was the first method we tried, and it worked well enough that we did not pursue other strategies. It may be that there exists a better method of integrating the different signals (perhaps a neural network or some other classifier), but this will have to be examined in future versions. The 4.25 and 0.4 constants were arrived at by experimenting with different values and observing the change in results across our test set of genomes. We chose the values such that they maximized performance across the entire set. In order to rule out bias in *E. coli, B. subtilis*, and *P. aeruginosa*, we also verified that the same approximate constants maximized performance on our set of genomes with those three excluded.

False positive reduction is an important goal in Prodigal. In order to reduce the number of overall predictions, Prodigal modifies the above start weight (4.25) based on the length of the gene. In examination of numerous genomes, we determined that approximately 250 bp is the point of equilibrium at which a gene with a positive coding score is equally likely to be a false positive or a true prediction. Genes less than 250 bp are therefore penalized according to their length divided by 250. If the start score is greater than 0, it is reduced to *l*/250*s, where *l *is the length of the gene. If the start score is less than 0, it is instead multiplied by 250/l*s. Finally, for all genes with negative coding scores, regardless of length, the start score is penalized by a small amount to prevent genes with moderately good start scores but bad coding scores from drifting above zero.

Once the scores have been calculated, the dynamic programming is performed a second time, using the more detailed node scores described above for the gene connections. For intergenic connections, operon distance provides a stronger weight in the second pass of dynamic programming. When two genes overlap by 1 or 4 bp, if the second gene lacks an RBS and has a negative RBS score, the requirement of an RBS is lifted and the score is increased to 0. In addition, the program adds small bonuses for distances less than 60 bp, and small penalties for distances greater than 180 bp. These distances correspond roughly to observed operon distances [16]. Although dynamic programming has order *n *log *n*, we limit the valid connections by distance, such that "long" connections can only be made between the start of a really long gene and its stop codon. The end result is that Prodigal must make a connection generally within 5 kb, so that it must choose a gene in this region, even if its score is negative. When the dynamic programming is complete, however, the program makes a final sweep through the models and removes any such genes with negative scores. In addition, the algorithm makes one final improvement to start calls that proved to be significant in our test set. When two starts are separated by a distance of less than 15 bp (determined empirically from our test set), Prodigal sets the coding of the two choices to be equivalent and uses only the start score (based on RBS motif and start type) to determine which start to choose for the final gene prediction.

**Figure 2:**
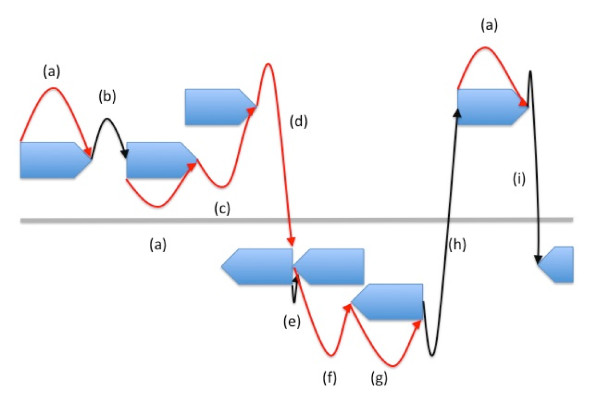
**Illustration of the dynamic programming connections in Prodigal**. The red arrows represent gene connections, and the black arrows represent intergenic connections. (a) 5' forward to 3' forward: Gene on the forward strand. (b) 3' forward to 5' forward: Intergenic space between two forward strand genes. (c) 3' forward to 3' forward: Overlapping genes on the forward strand. (d) 3' forward to 5' reverse: Forward and reverse strand genes whose 3' ends overlap. (e) 5' reverse to 3' reverse: Intergenic space between two reverse strand genes. (f) 3' reverse to 5' reverse: Gene on the reverse strand. (g) 3' reverse to 3' reverse: Overlapping genes on the reverse strand. (h) 5' reverse to 5' forward: Intergenic space between two opposite strand genes. (i) 3' forward to 3' reverse: Intergenic space between two opposite strand genes.

The final output of Prodigal consists of a complete list of gene coordinates and, at the user's specification, protein translations and/or detailed information about each potential start in the genome. Prodigal can be run either in two steps, with a training phase and a gene prediction phase, or in a single step where the training is hidden from the user and only the final genes are printed. A complete description of the algorithm in pseudocode can be found in Figure 2.

Prodigal runs very quickly, analyzing a 4 MB genome in about 20 seconds on a typical workstation. It is also extremely easy to use relative to other methods, consisting of only a single executable that can be run without the user needing to supply any organism-specific parameters. A web server has also been implemented at http://compbio.ornl.gov/prodigal/. The latest source code for Prodigal is available via the same web site, and version 1.20 has been included as an additional file [Additional File 1].

## Results and Discussion

Assessing the performance of microbial gene-finding programs remains a difficult task due to the lack of experimentally verified gene start sets. The EcoGene Verified Protein Starts set [12] remains the only large set of experimentally verified genes and translation start sites for typical bacteria. In addition, another large set was produced for two archaea, *Halobacterium salinarum *and *Natronomonas pharaonis*, using a special proteomics technique for extracting N-terminal peptides [17]. In addition to the above sets, numerous smaller sets exist for other genomes, but most of these are also atypical genomes such as cyanobacteria and archaea. Nonetheless, these still provide a set of experimentally verified genes with which to test the accuracy of start site and gene predictions. For these genomes, we relied on the data set from the ProTisa database of confirmed translation initiation sites [18]. However, some of the genes in the *Synechocystis *set were inconsistent with annotations in the Genbank files, and subsequent manual inspection proved the Genbank files to be correct. Therefore, we removed genes from this set, as well as a few genes from the other genomes, that disagreed with the Genbank annotations. We extracted all sets with more than 50 experimentally determined translation initiation sites, although we excluded numerous relatives of *E. coli *(and other genomes) in ProTisa whose starts were verified only through similarity search.

For purposes of assessing gene prediction quality, a gene call was considered correct if the algorithm identified the 3' end of the experimentally verified gene. Table 3 shows the performance of Prodigal relative to the programs GeneMarkHMM [2], Glimmer 3 [1], Easygene 1.2 [4], and MED 2.0 [5]. GeneMark [2] and Glimmer [1] predictions for these genomes were downloaded from NCBI [ftp://ncbi.nih.gov/genomes/Bacteria]. Easygene [4] predictions were obtained from the Easygene server. MED predictions were run locally using default parameters. The second number for each program indicates exactly correct genes (where both the translation initiation site and the stop codon are correctly identified). To assess the quality of the start site correction programs TiCo [7] and TriTisa [8], we chose to run these programs as postprocessors to Prodigal. Although this is different from the published results for these programs (which were applied to the final Genbank genes), we view this method as a more accurate way of assessing the *ab initio *value of such tools in an annotation pipeline.

**Table 3:** Gene Prediction Performance

Organism	%GC	Verified	Prodigal 1.20	Prodigal 1.20+TriTisa	Prodigal 1.20+TiCo	GeneMarkHMM 2.6	EasyGene 1.2	Glimmer 3.02	MED 2.0
*Escherichia coli K12*	50.8	884	884/853 (100%/96.5%)	884/840 (100.0%/95.0%)	884/843 (100.0%/95.4%)	882/835 (99.8%/94.5%)	880/809 (99.5%/91.5%)	880/804 (99.6%/91.0%)	875/810 (99.0%/91.6%)

*Halobacterium salinarum*	68.0	550	549/533 (99.8%/96.9%)	549/525 (99.8%/95.5%)	549/520 (99.8%/94.6%)	548/510 (99.6%/92.7%)	544/494 (98.9%/89.8%)	549/478 (99.8%/86.9%)	531/418 (96.6%/76.0%)

*Natronomonas pharaonis*	63.4	321	320/314 (99.7%/97.8%)	320/314 (99.7%/97.8%)	320/313 (99.7%/97.5%)	321/307 (100%/95.6%)	314/300 (97.8%/93.5%)	320/304 (99.7%/94.7%)	315/265 (98.1%/82.6%)

*Bacillus subtilis*	43.5	148	148/144 (100%/97.3%)	148/145 (100.0%/98.0%)	148/144 (100.0%/97.3%)	147/145 (99.3%/98.0%)	144/139 (97.3%/93.9%)	144/140 (97.3%/94.6%)	146/142 (98.7%/96.0%)

*Aeropyrum pernix*	56.3	131	131/128 (100%/97.7%)	131/127 (100.0%/97.0%)	131/128 (100.0%/97.7%)	130/123 (99.2%/93.9%)	130/124 (99.2%/94.7%)	130/121 (99.2%/92.4%)	131/116 (100%/88.6%)

*Synechocystis PCC6803*	47.8	102	102/99 (100%/97.0%)	102/98 (100%/96.1%)	102/93 (100%/91.2%)	102/92 (100%/90.2%)	101/87 (99.0%/85.3%)	102/84 (100%/82.4%)	100/88 (98.0%/86.3%)

*Pseudomonas aeruginosa*	66.6	122	118/116 (96.7%/95.1%)	118/113 (96.7%/92.6%)	118/115 (96.7%/94.3%)	115/105 (94.3%/86.1%)	122/112 (100%/91.8%)	120/113 (98.4%/92.6%)	117/113 (95.9%/92.6%)

*Mycobacterium tuberculosis H37Rv*	65.6	62	62/58 (100%/93.6%)	62/58 (100%/93.6%)	62/57 (100%/91.9%)	61/54 (98.4%/87.1%)	62/58 (100%/93.6%)	61/55 (98.4%/88.7%)	60/56 (96.8%/90.3%)

*Haemophilus influenzae*	38.2	67	67/66 (100%/98.5%)	67/67 (100%/100%)	67/67 (100%/100%)	67/65 (100%/97.0%)	67/67 (100%/100%)	67/65 (100%/97.0%)	66/65 (98.5%/97.0%)

*Sulfolobus solfataricus*	35.8	56	56/51 (100%/91.1%)	56/49 (100%/87.5%)	56/49 (100%/87.5%)	56/48 (100%/85.7%)	56/51 (100%/91.1%)	56/49 (100%/87.5%)	56/50 (100%/89.3%)

All Genomes	---	2443	2437/2362 (99.8%/96.7%)	2437/2336 (99.8%/95.6%)	2437/2329 (99.8%/95.3%)	2429/2284 (99.4%/93.5%)	2420/2241 (99.1%/91.7%)	2429/2213 (99.4%/90.6%)	2397/2123 (98.1%/86.9%)

Table 3 shows the performance of gene-finding algorithms on ten sets of experimentally verified genes with experimentally verified translation initiation sites. The first number in each entry indicates the number of 3' ends of genes correctly identified. The second number in each entry indicates the number of 5'+3' ends (genes and their correct starts) exactly identified. Beneath these numbers are % representations for each of those values. The final row shows the performance over the entire set of organisms.

As can be seen in Table 3, Prodigal proved equal or better at locating genes in every organism with a few exceptions: Glimmer 3 [1] and EasyGene [4] in *P. aeruginosa*, and GenemarkHMM [2] in *N. pharaonis*. Prodigal also performed equal to or better than the other tools in translation initiation site prediction with a few exceptions: GenemarkHMM [2] and TriTisa [8] on *B. subtilis*, and TiCo [7], TriTisa [8], and EasyGene [4] on *Haemophilus influenzae*. Prodigal performs equal or better at locating existing genes, while also providing comparable performance in translation initiation site prediction to the start correction tools.

The above test set contains many unusual genomes. *Bacillus subtilis *is remarkable for its extremely strong use of the SD motif. *Halobacterium salinarum *and *Natronomonas pharanois *make only limited use of the SD motif. *Aeropyrum pernix *contains a different RBS motif in GGTG. *Synechocystis PCC6803*, like most cyanobacteria, does not seem to use the SD motif at all, and instead favors AT-rich regions upstream of its translation start sites. A quick scan of 700 finished genomes in Genbank with Prodigal's SD-motif routine revealed that 88% of them used the SD motif. It is our assertion that *E. coli, B. subtilis*, and *P. aeruginosa *from the table above provide a more typical look at performance in the vast majority of sequenced microbial genomes.

Although Genbank files doubtless contain many errors, results for these same organisms vs. Genbank annotations were recorded to capture a "whole genome" view. Table 4 shows these results. All the genomes in table 4 have sets of more than 100 experimentally verified protein starts (in table 3). Although it is likely that they are still far from perfect, it is an interesting result nonetheless that Prodigal performed very well compared to all existing methods. Prodigal's performances on the well-studied cyanobacteria *Synechocystis PCC6803 *and the highly curated *Pseudomonas aeruginosa *are particularly interesting, in that the program matches many more start sites in the Genbank file than the other methods.

**Table 4:** Comparison with Genbank Annotations

Organism	Genbank Genes with no Joins	Prodigal 1.20	Prodigal 1.20+TiCo	Prodigal 1.20+TriTisa	GenemarkHMM 2.6	Glimmer 3.02	EasyGene 1.2	MED 2.0
*Escherichia coli K12*	4268	4118/3823 (96.5%/89.6%)	4118/3779 (96.5%/88.5%)	4118/3778 (96.5%/88.5%)	4122/3685 (96.6%/86.3%)	4076/3563 (95.5%/83.5%)	3977/3565 (93.2%/83.5%)	4102/3711 (96.1%/86.9%)

*Halobacterium salinarum*	2110	2062/1857 (97.7%/88.0%)	2062/1809 (97.7%/85.7%)	2061/1790 (97.6%/84.8%)	2042/1676 (96.7%/79.4%)	2054/1609 (97.3%/76.2%)	2018/1692 (95.6%/80.2%)	2008/1469 (95.1%/69.6%)

*Natronomonas pharaonis*	2661	2630/2398 (98.8%/90.1%)	2630/2358 (98.8%/88.6%)	2630/2348 (98.8%/88.2%)	2624/2251 (98.6%/84.6%)	2622/2220 (98.5%/83.4%)	2548/2271 (95.7%/85.3%)	2586/1953 (97.2%/73.4%)

*Bacillus subtilis*	4174	4113/3705 (98.5%/88.8%)	4113/3678 (98.5%/88.1%)	4113/3679 (98.5%/88.1%)	4136/3713 (99.1%/89.0%)	4102/3569 (98.3%/85.5%)	3977/3578 (95.3%/85.7%)	4127/3596 (98.9%/86.2%)

*Aeropyrum pernix*	1699	1670/1430 (98.3%/84.2%)	1670/1363 (98.3%/80.2%)	1670/1353 (98.3%/79.6%)	1672/1364 (98.4%/80.3%)	1671/1317 (98.4%/77.5%)	1652/1389 (97.2%/81.8%)	1689/1309 (99.4%/77.1%)

*Synechocystis PCC6803*	3171	3146/2587 (99.2%/81.6%)	3146/2364 (99.2%/74.6%)	3146/2447 (99.2%/77.2%)	3124/2337 (98.5%/73.7%)	3123/2236 (98.5%/70.5%)	3053/2288 (96.3%/72.2%)	3126/2192 (98.6%/69.1%)

*Pseudomonas aeruginosa*	5565	5514/5038 (99.1%/90.5%)	5514/4885 (99.1%/87.8%)	5514/4821 (99.1%/86.6%)	5484/4698 (98.5%/84.4%)	5491/4705 (98.7%/84.5%)	5522/4761 (99.2%/85.5%)	5292/4539 (95.1%/81.6%)

Table 4 shows the performance of gene-finding algorithms on seven Genbank files. The first number in each entry indicates the number of 3' ends of genes correctly identified. The second number in each entry indicates the number of 5'+3' ends (genes and their correct starts) exactly identified. Beneath these numbers are % representations for each of those values. It should be noted that Genbank genes are not experimentally verified; this table is just meant to provide a snapshot of performance over entire genomes.

These results cannot be seen as definitive, however, as it is always possible Prodigal's algorithm contains a bias that is shared by whatever methods were used to create the original Genbank files. In order to rule this bias out, we examined ATG usage and "leftmost start" usage in each of the methods, but we could find no obvious bias shared by Prodigal and Genbank annotations relative to the other methods. If anything, Prodigal seemed to call more starts internally and to truncate more genes, than the other methods. Although the quality of the Genbank files is impossible to estimate, we included the above results to demonstrate the concept of "genome-wide" performance, an important factor in microbial annotation pipelines.

Determining the number of false positives for each method is an impossible task without knowing the complete set of protein coding genes for each genome. Instead, we can only measure the number of genes predicted by each program relative to those retained by manual curators in the Genbank files. Table 5 shows the number of genes predicted by each program; the number in parentheses is normalized around the number of genes in the Genbank file.

**Table 5:** Number of Genes Predicted By Each Method

Organism	Genbank	EasyGene 1.2	Prodigal 1.20	GenemarkHMM 2.6	Glimmer 3.02	MED 2.0
*Escherichia coli K12*	4321 (1.00)	4099 (0.95)	4305 (1.00)	4378 (1.01)	4476 (1.04)	4811 (1.11)

*Halobacterium salinarum*	2110 (1.00)	2097 (0.99)	2101 (1.00)	2085 (0.99)	2141 (1.01)	2385 (1.13)

*Natronomonas pharaonis*	2661 (1.00)	2587 (0.97)	2678 (1.01)	2685 (1.01)	2720 (1.02)	3111 (1.17)

*Bacillus subtilis*	4177 (1.00)	4019 (0.96)	4224 (1.01)	4354 (1.04)	4429 (1.06)	4601 (1.10)

*Aeropyrum pernix*	1700 (1.00)	1686 (0.99)	1717 (1.01)	1738 (1.02)	1789 (1.05)	2419 (1.42)

*Synechocystis PCC6803*	3172 (1.00)	3089 (0.97)	3306 (1.04)	3462 (1.09)	3677 (1.16)	3778 (1.19)

*Pseudomonas aeruginosa*	5566 (1.00)	5910 (1.06)	5679 (1.02)	5712 (1.03)	5878 (1.06)	6709 (1.21)

Table 5 shows the number of genes predicted by each method on seven Genbank files. The Genbank column indicates the number of genes in the Genbank file. The number in parentheses indicates the number of predicted genes divided by the number of genes in the Genbank file, e.g. 1.10 indicates 10% more genes predicted than the Genbank file.

EasyGene [4] predicts fewer genes than all other methods on every genome, with the one exception of *Pseudomonas aeruginosa*; however, Easygene [4] is also less sensitive than the other programs (as can be seen in tables 3 and 4). It is likely the program could be improved on these genomes simply by using a less stringent R-value threshold, though this would lead to an increase in the number of genes predicted. Prodigal predicts equal or fewer genes vs. the remaining methods (excepting EasyGene) in all cases except *Halobacterium salinarum *vs. Genemark [2], while still retaining excellent sensitivity in locating genes. The gaps in *B. subtilis *and *Synechocystis *are particularly noticeable.

In the future, we hope to improve Prodigal's recognition of short genes, atypical genes, translation initiation mechanisms, and genomes. With a more detailed look at cyanobacteria and archaea, in general, it should be possible to build a better start site prediction algorithm than the one currently in place for non-SD motifs. Also, identifying laterally transferred genes, genes in phage regions, proteins with signal peptides, and any other genes that do not match the typical GC frame bias for the organism in question, are areas where Prodigal can improve. We will also seek to develop a version of Prodigal to address the rapidly growing metagenomic data for microbial organisms.

## Conclusions

We developed a new gene-finding program for microbial genomes called Prodigal. The goals of Prodigal were to attain greater sensitivity in identifying existing genes, to predict translation initiation sites more accurately, and to minimize the number of false positive predictions. The results of Prodigal were compared to existing methods for both purely experimentally verified genes as well as curated Genbank files for a number of genomes. Prodigal's performance was found to be comparable or better to existing methods in the prediction of genes while also predicting fewer overall genes. In the prediction of translation initiation sites, Prodigal performed competitively with existing methods. Prodigal is currently already in use at many institutions, and it has been used to annotate all finished microbial genomes submitted to Genbank by DOE-JGI in 2008 and onward (a substantial percentage of the overall finished microbial genomes at NCBI). It is run regularly at NCBI alongside GenemarkHMM [2] and Glimmer [1], and it has also been incorporated into the Swiss Institute of Bioinformatics microbial genomics browser [19]. In conclusion, Prodigal should prove to be a valuable resource for genome annotation of either draft or finished microbial sequence.

## Availability and Requirements

**Project Name**: Prodigal

**Project Home Page**: http://compbio.ornl.gov/prodigal/

**Operating System**: Any

**Programming Language**: C

**License**: GNU GPL

## Authors' contributions

DH wrote the code. DH and LH designed the algorithm. PL and GLC examined the GC frame plot problem in high GC genomes. GLC also performed a study on 16s RNAs. ML and FL contributed ideas and suggestions to the algorithm, as well as practical testing of the code. All authors read and approved the final manuscript.

## Supplementary Material

Additional file 1 **prodigal.v1_20.tar.gz**. Archive containing the source code for Prodigal. Click here for file

## References

[B1] Delcher A Bratke K Powers E Salzberg S Identifying bacterial genes and endosymbiont DNA with Glimmer Bioinformatics 2007 23 6 673 679 10.1093/bioinformatics/btm009 17237039 PMC2387122

[B2] Lukashin A Borodovsky M GeneMark.hmm: new solutions for gene finding Nucleic Acids Res 1998 26 4 1107 1115 10.1093/nar/26.4.1107 9461475 PMC147337

[B3] Benson D Karsch-Mizrachi I Lipman D Ostell J Sayers E GenBank Nucleic Acids Res 2009 37 Database D26 31 10.1093/nar/gkn723 18940867 PMC2686462

[B4] Larsen T Krogh A EasyGene--a prokaryotic gene finder that ranks ORFs by statistical significance BMC Bioinformatics 2003 4 21 10.1186/1471-2105-4-21 12783628 PMC521197

[B5] Zhu H Hu G Yang Y Wang J She Z MED: a new non-supervised gene prediction algorithm for bacterial and archaeal genomes BMC Bioinformatics 2007 8 97 10.1186/1471-2105-8-97 17367537 PMC1847833

[B6] Ou H Guo F Zhang C GS-Finder: a program to find bacterial gene start sites with a self-training method Int J Biochem Cell Biol 2004 36 3 535 544 10.1016/j.biocel.2003.08.013 14687930

[B7] Tech M Pfeifer N Morgenstern B Meinicke P TICO: a tool for improving predictions of prokaryotic translation initiation sites Bioinformatics 2005 21 17 3568 3569 10.1093/bioinformatics/bti563 15994191

[B8] Hu G Zheng X Zhu H She Z Prediction of translation initiation site for microbial genomes with TriTISA Bioinformatics 2009 25 1 123 125 10.1093/bioinformatics/btn576 19015130

[B9] Altschul S Gish W Miller W Myers E Lipman D Basic local alignment search tool J Mol Biol 1990 215 3 403 410 2231712 10.1016/S0022-2836(05)80360-2

[B10] VerBerkmoes N Shah M Lankford P Pelletier D Strader M Tabb D McDonald W Barton J Hurst G Hauser L Determination and comparison of the baseline proteomes of the versatile microbe Rhodopseudomonas palustris under its major metabolic states J Proteome Res 2006 5 2 287 298 10.1021/pr0503230 16457594

[B11] Badger J Olsen G CRITICA: coding region identification tool invoking comparative analysis Mol Bio Evol 1999 16 4 512 24 10.1093/oxfordjournals.molbev.a026133 10331277

[B12] Rudd K EcoGene: a genome sequence database for Escherichia coli K-12 Nucleic Acids Res 2000 28 1 60 64 10.1093/nar/28.1.60 10592181 PMC102481

[B13] Rutherford K Parkhill J Crook J Horsnell T Rice P Rajandream M Barrell B Artemis: sequence visualization and annotation Bioinformatics 2000 16 10 944 5 10.1093/bioinformatics/16.10.944 11120685

[B14] Bellman R On the Theory of Dynamic Programming Proc Natl Acad Sci USA 1952 38 8 716 719 10.1073/pnas.38.8.716 16589166 PMC1063639

[B15] Shine J Dalgarno L Terminal-sequence analysis of bacterial ribosomal RNA. Correlation between the 3'-terminal-polypyrimidine sequence of 16-S RNA and translational specificity of the ribosome Eur J Biochem 1975 57 1 221 230 10.1111/j.1432-1033.1975.tb02294.x 809282

[B16] Dam P Olman V Harris K Su Z Xu Y Operon prediction using both genome-specific and general genomic information Nucleic Acids Res 2007 35 1 288 98 10.1093/nar/gkl1018 17170009 PMC1802555

[B17] Aivaliotis M Gevaert K Falb M Tebbe A Konstantinidis K Bisle B Klein C Martens L Staes A Timmerman E Large-scale identification of N-terminal peptides in the halophilic archaea Halobacterium salinarum and Natronomonas pharaonis J Proteome Res 2007 6 6 2195 2204 10.1021/pr0700347 17444671

[B18] Hu G Zheng X Yang Y Ortet P She Z Zhu H ProTISA: a comprehensive resource for translation initiation site annotation in prokaryotic genomes Nucleic Acids Res 2008 36 Database D114 119 17942412 10.1093/nar/gkm799 PMC2238952

[B19] Gattiker A Dessimoz C Schneider A Xenarios I Pagni M Rougemont J The Microbe browser for comparative genomics Nucleic Acids Res 2009 37 Web server W296 9 10.1093/nar/gkp268 19406928 PMC2703916

